# Hopeless tooth? Prognosis and comprehensive treatment. A case report

**DOI:** 10.25122/jml-2021-0080

**Published:** 2021

**Authors:** Mihaela Maria Grigorie, Ioana Suciu, Daniela Zaharia, Ecaterina Ionescu, Mihaela Chirila, Monica Voiculeanu

**Affiliations:** 1. Department of Endodontics, University of Medicine and Pharmacy Carol Davila, Faculty of Dental Medicine, Bucharest, Romania; 2. Private Practice, Bucharest, Romania

**Keywords:** periodontal regeneration, combined regeneration techniques, regenerative potential of periodontium, enamel matrix derivatives, ortho-perio treatment

## Abstract

A hopeless tooth from a periodontal point of view, with severe bone resorption, mobility and abnormal tooth migration, is often extracted. In advanced cases, function and esthetics are impaired, and an interdisciplinary treatment is requested. Retaining or not these teeth is based on clinician judgment. A growing body of evidence claims that prognosis has great potential to be improved in a motivated patient with good oral hygiene and regular maintenance. This case report aims to present a periodontal regenerative technique combining enamel matrix protein derivatives and a particulated xenograft to treat intraosseous defects caused by periodontitis. The patient healed uneventfully, and no complications were recorded after the surgical procedure. To correct abnormal tooth migration and improve function and esthetics, orthodontic treatment was instituted. Tooth prognosis improved from hopeless to questionable. This approach extended the life span of a compromised tooth, improving periodontal support and decreasing tooth mobility. This could be an alternative to extraction and implant.

## Introduction

Periodontitis is a chronic inflammatory disease of infectious origin that causes progressive deterioration and destruction of alveolar bone, periodontal ligament and radicular cement [1]. The extent and severity of bone loss must be diagnosed with radiographs and clinical examination [2]. In periodontitis, there are two types of bone loss: horizontal and vertical [3]. The vertical type involves a lesion placed apical to the residual alveolar bone and is more susceptible to regeneration techniques [2, 4, 5].

Teeth with deep pockets associated with deep vertical defects have poor treatment predictability and, according to literature, are considered hopeless and more often are deemed to extraction [6–8]. A considerable number of teeth with no or minor coronal destruction were extracted at an attachment level of 50–70%. The threshold for ‘periodontal’ extractions seems to be too low and undifferentiated [9]. Changing the prognosis of these teeth would avoid extraction and replacement with an implant.

The American Academy of Periodontology (AAP) defines periodontal regeneration as the restoration of tissue loss due to periodontitis, including radicular cement, periodontal ligament, and alveolar bone [1]. Periodontal regeneration is possible in intraosseous defects [10, 11]; it could be obtained using guided tissue regeneration (GTR), enamel matrix proteins (EMP), or bone grafts (BG). GTR consists of placing a biocompatible membrane between the epithelium and connective tissue of the defect, which can serve as a physical barrier, promoting the migration of cells from the periodontal ligament and impeding the entry of epithelial cells [12, 13]. EMPs, extracted from the embryonic enamel of young pigs, are a gel material placed inside the defect, promoting proper periodontal regeneration. EMPs simulate events that occur during the formation of the root and promote the formation of new alveolar bone, radicular cement, and periodontal ligament. EMPs have antimicrobial abilities and inhibit epithelial migration by direct contact [4, 14, 15]. The most widely used BGs in periodontal regeneration are bone xenografts extracted from lyophilized bone, usually of bovine origin.

It has been demonstrated that BGs alone are ineffective in achieving satisfactory results, so they are used in combination with GTR and EMP. The combination of EMP and BG, as well as GTR and BG, shows additional improvement in the reduction of pocket depth and gains in clinical attachment versus EMP or membranes alone [10, 12, 16].

Reduction of periodontal support in severe cases of periodontitis is associated with abnormal tooth migration. Advanced periodontal disease may create infrabony defects adjacent to pathologically migrated teeth. In order to recreate esthetics and function, the use of combined orthodontic-periodontic treatment is required [17].

## Case Report

**Figure 1 AB.**
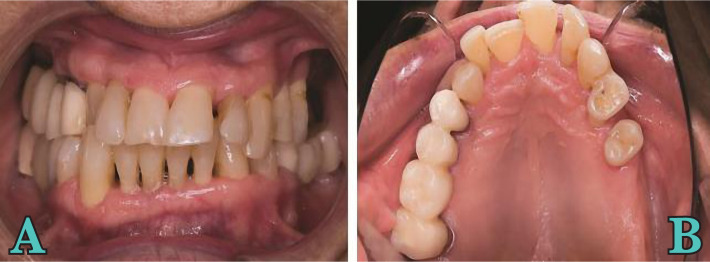
Initial clinical view.

In 2016, a 62-year-old caucasian female presented at a private dental office with a good general health condition; her main complaint was related to changes in the position of upper and lower teeth, mobilities, and gingival bleeding during brushing (Figure 1 AB).

She was a non-smoker and did not have chronic marginal periodontitis. Dental history included: dental visits yearly, dental extractions due to caries, endodontic treatments, and fixed prostheses. Daily oral hygiene routine included brushing 2 times a day for 2 min with a manual medium bristles toothbrush without any attempt to clean interdental spaces. After periodontal and full series radiographic exam, and picture-taking, the patient was diagnosed with generalized severe chronic periodontitis [18], stage IV, grade B, based on the current classification proposed by Tonetti *et al.* in 2018 [19], with the presence of vertical defects in some teeth, furcation involvement, mobilities, as well as supra- and subgingival plaque deposits and abnormal tooth migration. Tooth prognosis was evaluated at baseline and re-evaluated at the 1- year and 4-year examination applying the score proposed by Kwok and Caton in 2007 [20]. Their score goes from “favorable” to “questionable” to “unfavorable” to “hopeless”.

### Treatment methods

**Figure 2.**
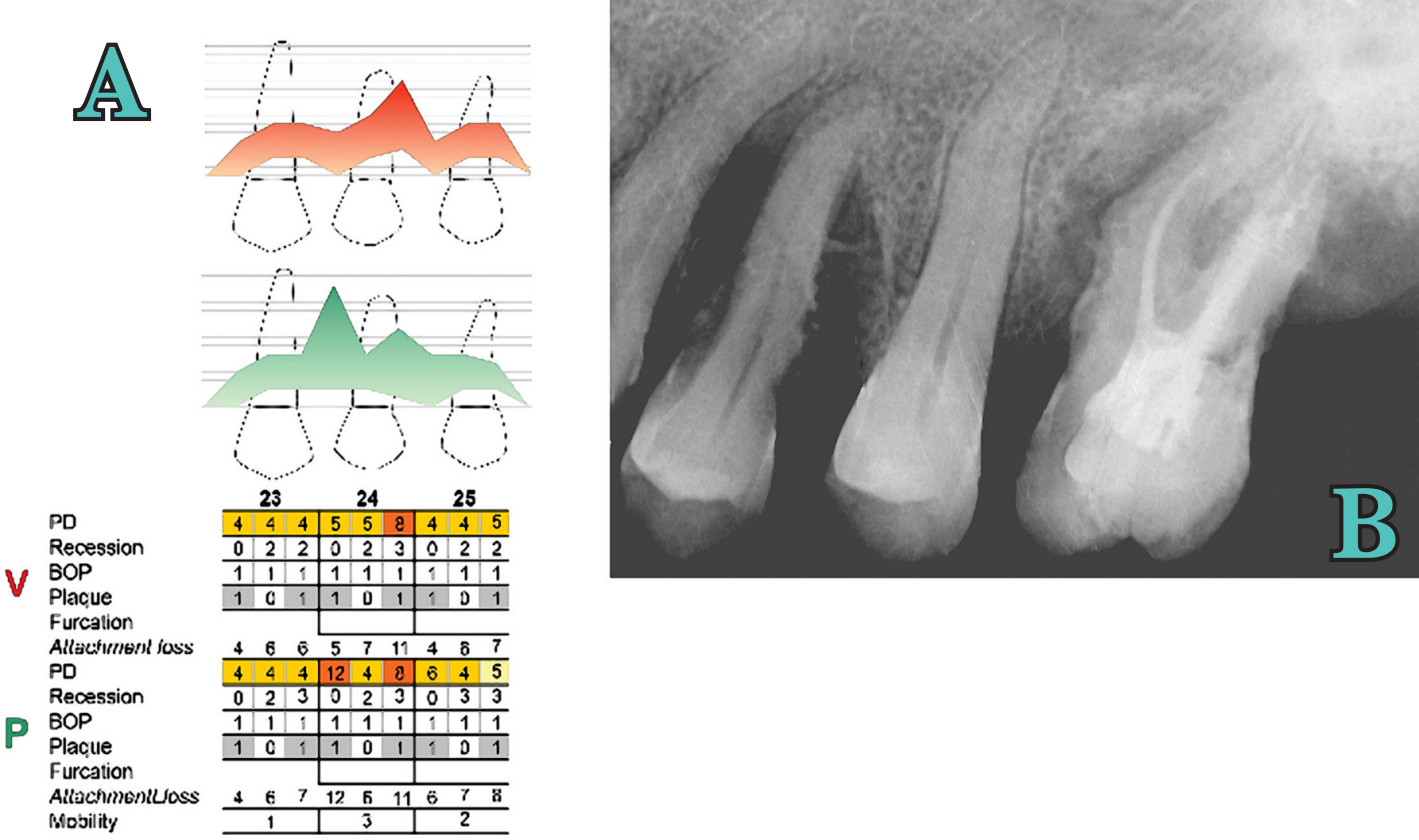
Initial periodontal (A) and radiographic examination (B).

**Figure 3 AB.**
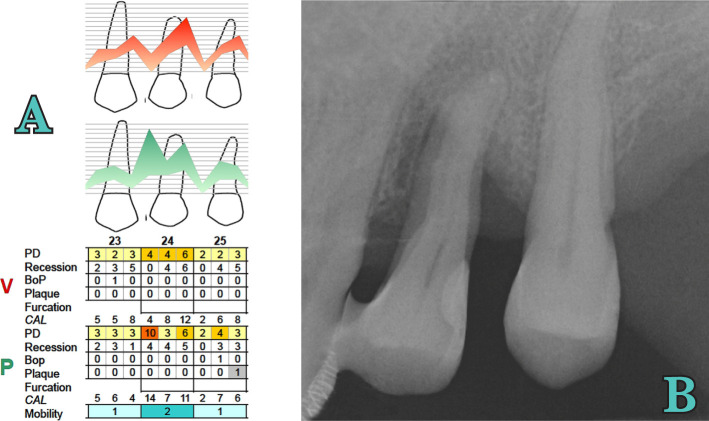
X-ray at 3 months from the initial examination - periodontal (A) and radiographic examination (B).

**Figure 4.**
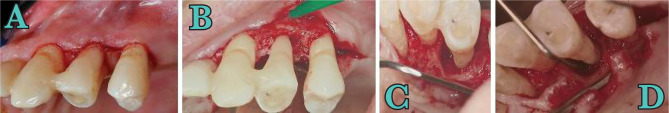
A) Incision; B) Flap raised; C)Bone defect; D) Depth of bone defect.

**Figure 5.**
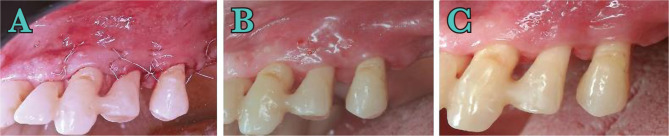
A) Suture; B) Suture removal at 2 weeks; C) 1 month after surgery.

The comprehensive therapy that we adopted included four important steps:

1. Initial therapy: Scaling and root planing in 4 sessions, occlusal adjustment, splinting of mobile teeth, and oral hygiene instructions were given to the patient in the initial phase. Clinically and radiographically, tooth 2.4 was vital, extruded, presented fremitus, an important loss of periodontal support close to the apex, no furcation involvement, and grade 3 mobility was noted [21] (Figure 2). At the 3-month re-evaluation, full-mouth plaque score (FMPS) and full-mouth bleeding score (FMBS) were 15%, tissues were contracted, pocket depth reduced, but remained deep around some teeth, and dental mobility was improved. One tooth was extracted due to caries and endodontic complications. At 3 months, tooth 2.4 responded positive to the vitality test and had a deep palatal probing depth of 10 mm mesial and 6 mm distal. A remarkable improvement in bone mineralization was observed on x-ray compared with the initial one (Figure 3AB). Periodontal regenerative surgery was decided to be appropriate for this tooth. The surgical phase of treatment was formulated. Informed consent was obtained from the patient after careful explanation of the surgical procedure, prognosis, and possible complications.
2. Surgical procedure: After assessment of the clinical variables and local anesthesia, a flap was prepared. Considering the anatomical defect and the fact that interproximal space was >2 mm, a modified papilla preservation technique (MPPT) was decided with a full-thickness vestibular incision at the base of the papilla on 2.2, 2.3, 2.4, 2.5 extended in crest distally on 2.5. Following full-thickness flap elevation, the granulation tissue was removed using Gracey metal curettes (HuFriedy®, Chicago, IL, USA) (Figure 4AB). Scaling and root planing were performed by combining the use of metal curettes and power-driven instrumentation through an ultrasonic scaler (EMS®, Nyon, Switzerland). An 11-mm wide vertical defect was present on the mesial palatal side of 2.4 and 7 mm distally (Figure 4 CD). Root conditioning with a 24% EDTA gel (PrefGel®, Institute Straumann, Switzerland) was performed. Immediately after root conditioning, EMD and BioOss were applied, followed by a tension-free primary closure of the interdental papillae and of the mucoperiosteal flaps utilizing 6-0 and 5-0 monofilament (PTFE) non-resorbable suturing material (Profimed, Medipac® Greece) (Figure 5A).

### Post-surgical instructions and infection control

Pain and edema were controlled with 600 mg ibuprofen immediately before the surgical intervention and after 4 hours. Subjects were instructed to rinse twice daily with 0.12% chlorhexidine digluconate (Curasept ADS® Curaden AG, Kriens, Switzerland) for the first 2 weeks and use modified oral hygiene procedures in the treated areas for the first four postoperative weeks. Also, systemic antibiotics were prescribed. After 4 weeks, subjects were instructed to resume regular self-performed oral hygiene procedures. Sutures were removed after 14 days (Figure 5B), and the patient was recalled after 4 weeks (Figure 5) and after 3, 6, 9, and 12 months for professional maintenance care. After 12 months, a follow-up examination was performed. In this case, the pocket examination was not performed 12 months after surgery.

### Follow-up

**Figure 6.**
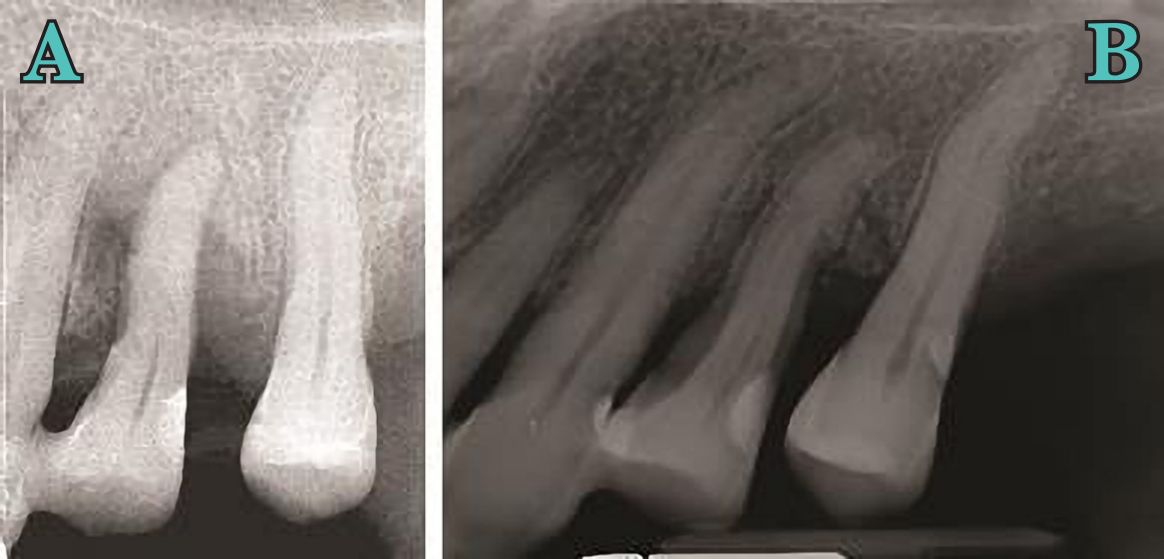
Tooth 2.4 x-ray at 3 months from the initial examination - periodontal (A) and radiographic examination (B).

After the surgery, the patient was examined weekly for the first month, and prophylaxis of the area was carried out at each visit. Brushing was suspended for 4 weeks. After one month, healing was satisfactory. After the first month, follow-up visits were scheduled every 3 months, emphasizing the importance of good hygiene, especially at the interproximal level. A detailed re-evaluation was performed one year after regenerative surgery, and a periapical x-ray revealed radiographic bone fill of almost the entire defect (Figure 6B. Clinically, the tooth is vital, and the pocket revealed 4 mm mesiopalatal with a 4 mm recession and 3 mm distopalatal with a 6 mm recession.

**Figure 7.**
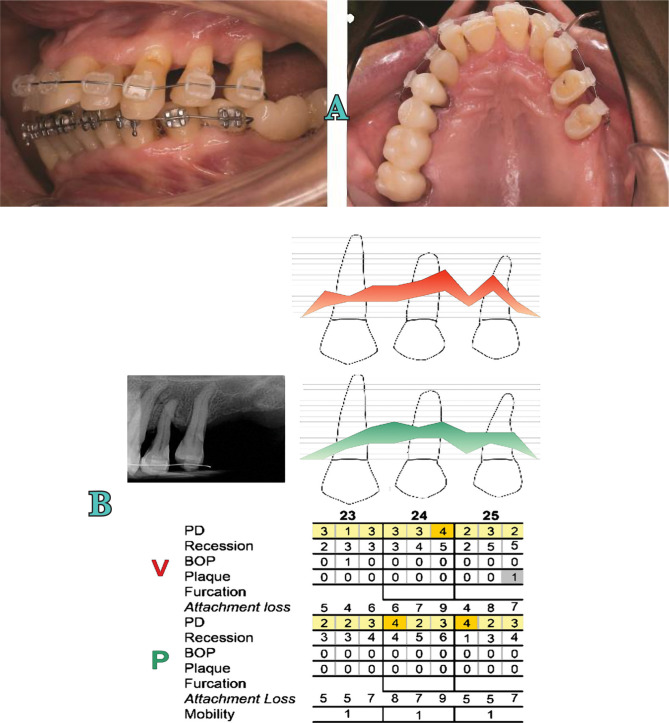
Orthodontic treatment. A) Clinical image; B) Rradiographic and periodontal examination.

**Figure 8.**
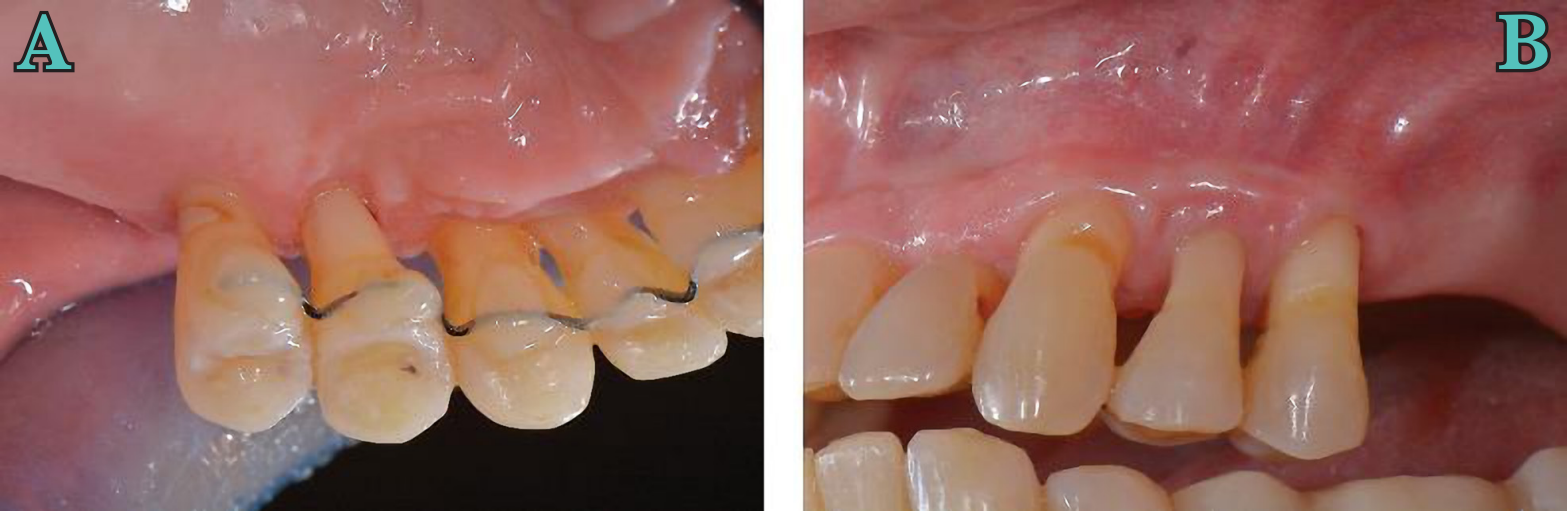
Palatal (A) and vestibular (B) view at 4 years after surgery.

**Figure 9.**
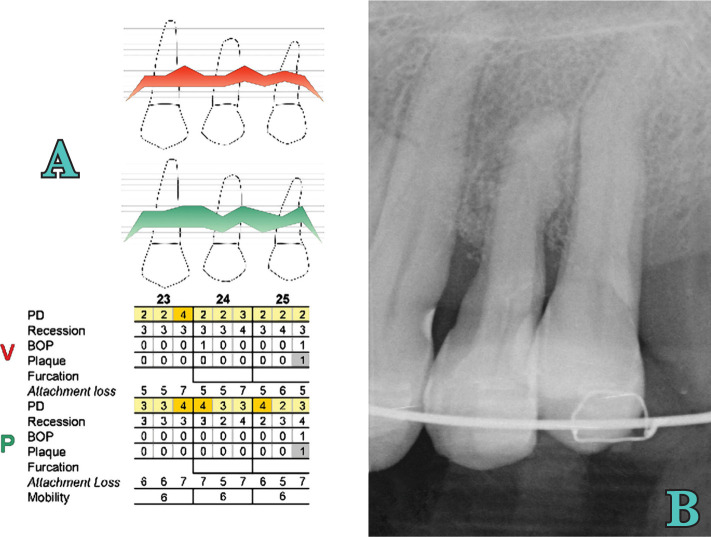
Periodontal (A) and radiographic (B) examination at 4 years.

3. Orthodontic therapy: One year after surgery, the patient was scheduled for orthodontic treatment to correct abnormal tooth migration and to improve function and esthetics (Figure 7AB). This treatment was administered for 1.5 years.
4. Supportive periodontal therapy: After the orthodontic therapy, regarding risk assessment, the patient was included in a maintenance program of Supportive Periodontal Therapy (SPT) every 3 months. Four years later, the tooth is vital and had a reduced but healthy periodontal tissue around (Figure 8 AB, Figure 9 AB).

### Treatment results

The tooth healed uneventfully after the surgical procedure, and no adverse effects, infections, or incomplete flap closure were noted with the grafting materials. At the end of the orthodontic treatment, the tooth was moved mesially and intruded. Plaque control was good, and a slight inflammation was present in the fourth year. Initial and final clinical parameters are reported in Table 1.

**Table 1.** Clinical parameters of tooth 2.4 - baseline vs. 4-year comparison.

2.4 Deepest site	Baseline	1-year	Δ Baseline 1 year	4-years	Δ Baseline 4 years
**PPD (mm)**	12	4	8	4	8
**REC (mm)**	0	4	-4	3	-3
**CAL (mm)**	12	8	4	7	5

Δ, difference; CAL – clinical attachment level; PPD – probing pocket depth; REC – gingival recession.

At 1 year, the deepest interproximal site at baseline had a clinical attachment level (CAL) gain of 4 mm and a pocket-depth (PD) reduction of 8 mm. At 4 years, in the same site, PD reduction remained 8 mm, and CAL gain was 5 mm. Also, tooth mobility grade 1 was noted.

## Discussion

In this case, a tooth considered hopeless due to reduced periodontal support and the presence of an infrabony defect close to the root apex was treated with combined orthodontic-periodontal therapy. The defect was first regenerated with collagen bovine bone mineral and EMD and then moved orthodontically. After that, the patient was included in an SPT program.

In an animal study, Araujo *et al.* demonstrated that it is possible to move a tooth into an area of an alveolar ridge previously augmented with bovine bone mineral and that the augmented bone did not impede orthodontic tooth movement [22]. The results of combined periodontal-orthodontic treatment in infrabony defects were presented in several investigations [23, 24, 25]. A mean PD reduction of 4.35 mm was reported; it was associated with a mean CAL gain of 5.5 mm at 10 months after orthodontic treatment. According to the authors, PD improvement was due to intrusion, retraction, and mesialisation movements.

In the present case, the orthodontic movement started 1 year after surgical periodontal treatment on a tooth with a PD of 4 mm. No periodontal changes occurred after orthodontic movement – the PD reduction of 8 mm, and a CAL gain of 4 mm that were obtained after regenerative periodontal treatment remained stable after orthodontic treatment.

This significant PD reduction seems to be related to active periodontal therapy and not to orthodontic treatment. This supports the fact that orthodontic treatment of healthy but reduced periodontium could be performed without adverse effect, and the results may be maintained in time if adequate SPT is provided [26, 27]. In this case, clinical outcomes are in accordance with those published by Cortellini *et al.* in 2011 [28]. The authors reported an average CAL gain of 7.7±2.8 mm and a PD reduction of 8.8±3 mm after regenerative surgery. Radiographic measurements were not made in this case, but the final radiographic image revealed improvement of the vertical defect, with the resolution of horizontal and vertical components. Radiographic bone levels obtained after active periodontal treatment remained the same after 4 years.

According to Kwok and Caton, tooth prognosis changed from hopeless to questionable due to the presence of the interradicular groove on the mesial surface of the root [20].

The results demonstrated that periodontal regeneration is effective and may be considered an alternative to extraction in teeth with advanced loss of periodontal support.

## Conclusions

Periodontal regeneration is effective in the treatment of vertical bone defects, even in teeth with a poor prognosis. It is essential to know that orthodontic tooth movement has no substantial effect on the healthy but reduced periodontium.

Therefore, it is crucial to establish the need and consequences of the interdisciplinary periodontal-orthodontic approach in order to deliver the best results for the patient.

## Acknowledgments

### Consent to participate

Written informed consent was obtained from the participants.

### Conflict of interest

The authors declare that there is no conflict of interest.
